# Involvement of the TRPV1 channel in the modulation of spontaneous locomotor activity, physical performance and physical exercise-induced physiological responses

**DOI:** 10.1590/1414-431X20165183

**Published:** 2016-05-10

**Authors:** A.S.R. Hudson, A.C. Kunstetter, W.C. Damasceno, S.P. Wanner

**Affiliations:** Laboratório de Fisiologia do Exercício, Escola de Educação Física, Fisioterapia e Terapia Ocupacional, Universidade Federal de Minas Gerais, Belo Horizonte, MG, Brazil

**Keywords:** Afferent, Capsaicin, Hyperthermia, Metabolism, Physical activity, Thermoregulation

## Abstract

Physical exercise triggers coordinated physiological responses to meet the augmented metabolic demand of contracting muscles. To provide adequate responses, the brain must receive sensory information about the physiological status of peripheral tissues and organs, such as changes in osmolality, temperature and pH. Most of the receptors involved in these afferent pathways express ion channels, including transient receptor potential (TRP) channels, which are usually activated by more than one type of stimulus and are therefore considered polymodal receptors. Among these TRP channels, the TRPV1 channel (transient receptor potential vanilloid type 1 or capsaicin receptor) has well-documented functions in the modulation of pain sensation and thermoregulatory responses. However, the TRPV1 channel is also expressed in non-neural tissues, suggesting that this channel may perform a broad range of functions. In this review, we first present a brief overview of the available tools for studying the physiological roles of the TRPV1 channel. Then, we present the relationship between the TRPV1 channel and spontaneous locomotor activity, physical performance, and modulation of several physiological responses, including water and electrolyte balance, muscle hypertrophy, and metabolic, cardiovascular, gastrointestinal, and inflammatory responses. Altogether, the data presented herein indicate that the TPRV1 channel modulates many physiological functions other than nociception and thermoregulation. In addition, these data open new possibilities for investigating the role of this channel in the acute effects induced by a single bout of physical exercise and in the chronic effects induced by physical training.

## Introduction

The biomechanical and physiological characteristics of humans suggest that the ability to engage in endurance running was important for obtaining food prior to the adoption of a modern lifestyle (1). Endurance running is defined as running many kilometers over extended time using aerobic metabolism (1). During muscle contraction, several physiological responses occur in the human body, including the mobilization of energy substrates, activation of the sympathoadrenal system, an increase in the heart rate, and the redistribution of blood flow (2,3); collectively, these responses allow adaptation to increased energy expenditure. Because of these physiological responses, physical activity or its subset, physical exercise (i.e., the planned, structured, and repetitive physical activity), can be performed at metabolic rates that are 15- to 20-fold higher than the resting metabolic rate (4).

Physical exercise-induced physiological responses are controlled by homeostatic systems, which have principles of operation. Homeostatic systems generally use more than one effector because effector redundancy extends the range for controlling the monitored variables (5). This principle is evidenced by the fact that the lipolysis rate is increased in response to several hormones (e.g., adrenaline, cortisol, growth hormone) enables the compensatory activation of alternative effectors when one of these effectors fails or becomes incapacitated. Moreover, different homeostats (i.e., central integration systems stimulated by different afferent pathways) can regulate the activity of the same effector system. For instance, the regulation of plasma volume and electrolytic balance share the vasopressin effector (5). The neural pathways of homeostatic systems often consist of afferent and efferent components and a central integration system; these pathways are not fully described, particularly during exercise.

In the aforementioned context, central integration studies have identified the brain structures and neurotransmitters controlling exercise-induced physiological responses, and substantial progress has been achieved in this area (6–10). However, more research on the participation of afferent pathways in the modulation of physiological responses is still required. A fundamental aspect of homeostatic system function is that blocking the transmission of afferent information to a central integration system will increase the changeability of the monitored variables (5). In agreement with this concept, we demonstrated the fundamental role of arterial baroreceptor signaling in exercise-induced cardiovascular responses (11).

In a different perspective, the role of afferent pathways in exercise fatigue is still debated. Some authors state that the changes occurring at peripheral physiological systems, such as substrate depletion and changes in temperature or metabolite accumulation, act as afferent signalers, which modulate control processes in the brain in a dynamic, non-linear, and integrative manner (12). Nevertheless, there is not a consensus about this theoretical model that was proposed to explain exercise fatigue (13). Undoubtedly, a deeper understanding of the participation of afferent pathways in exercise-induced physiological responses will help elucidate whether afferents are in fact involved in fatigue and in the modulation of physical performance.

## Transient receptor potential (TRP) channels

Most of the receptors involved in sensorial, afferent pathways express ion channels that sense the physiological status of peripheral tissues, including changes in osmolality, pH, temperature and the presence of other chemical and physical factors. Evidence indicates that the ion channels termed transient receptor potential (TRP) channels are among the receptors responsible for sending sensorial information from the periphery to the central integration system. Six TRP subfamilies have been identified: canonical (TRPC1–7), vanilloid (TRPV1–6), melastatin-like (TRPM1–8), polycystin (TRPP2, TRPP3, and TRPP5), mucolipin (TRPML1–3), and ankyrin-rich (TRPA1) (14).

Recent studies have highlighted the fact that local temperature sensing is a function mediated by the activation of TRP channels (14–16). Eleven TRP channels are highly sensitive to temperature, with three of these channels being activated by cold (TRPM8, TRPA1 and TRPC5) and eight that are activated by heat (TRPV1–4 and TRPM2–5) (14). The thermosensitive TRP channels are activated in response to changes in the local temperature; each channel is sensitive to a specific temperature range. Acting together, these channels sense temperatures ranging from noxious heat to noxious cold (17). Importantly, many TRP channels are activated by stimuli other than temperature and are, therefore, considered polymodal receptors (18).

Among these polymodal TRP channels, the TRPV1 channel (transient receptor potential vanilloid type 1 or capsaicin receptor) has important physiological functions, including the modulation of pain sensation and the physiological responses induced by physical exercise. The TRPV1 channel was isolated and identified by Caterina et al. (19), and as shown *in vitro*, the channel is sensitive to a temperature range corresponding to noxious heat (activated by temperatures higher than 43°C). Aside from being activated by heat, the TRPV1 channel is also activated by changes in pH (20,21), by endogenous lipoxygenase products, such as anandamide and oleoylethanolamide (17,19,21), and by exogenous ligands known as vanilloids (e.g., capsaicin and resiniferatoxin; RTX) (20,21). The latter mode of activation was the reason to name these channels vanilloid receptors. Of note, capsaicin is the principal irritating and pungent constituent of various species of red peppers (22). When one of the aforementioned stimuli bind to specific sites of the TRPV1 molecule, this channel opens, causing a transient influx of ions, particularly calcium, and thereby promoting depolarization of the cell membrane (23).

The TRPV1 channel is mainly present in myelinated nerve fibers (type Aδ) and unmyelinated (type C) nociceptors, which have neural bodies in the dorsal root ganglia, trigeminal ganglia and nodose ganglia (19). TRPV1 expression has been found in the vagus nerve and in brain nuclei that receive vagal afferents, such as the nucleus of the solitary tract (24,25). The TRPV1 channel is also expressed in the hypothalamus (26), skeletal muscles (27) and other peripheral tissues, including the gastrointestinal tract (28), endothelial and smooth muscle cells (29) and arteries (30).

## Brief overview of the available tools for studying the physiological roles of the TRPV1 channel

The scientific literature presents different tools for investigating the physiological functions mediated by the TRPV1 channel. These tools can be divided into three major classes: genetic, nutritional and pharmacological.

The genetic tools have been used to create mice with a deletion of the *Trpv1* gene (31,32) and rats that under- or overexpress the TRPV1 channel (33,34). There are several uses for these genetic tools: for instance, knockout mice can be used as a model for human diseases and permit the exploration of the physiological function and significance of specific genes *in vivo* (35). Nevertheless, the negative results obtained from knockout mice are generally inconclusive because compensatory changes may restore the function of interest, even when the deleted gene is normally responsible for a given function (17). These methods are not applicable to humans. Related studies in humans associate the magnitude of a given response promoted by a nutritional or pharmacological stimulus with the genetic variations that exist within a population. For example, the genetic variant TRPV1 Val585Ile correlates significantly with the change in abdominal adiposity induced by the prolonged ingestion of capsinoids (36).

The nutritional tools involve the acute or chronic ingestion of capsaicin or non-pungent capsaicin analogs, such as capsiate. This method is employed in experiments with both animals and humans (37,38). However, care should be taken to avoid the direct translation of the outcomes observed in different species because mice, rats and humans have quite different daily food intakes and metabolic rates. Moreover, a researcher should be aware that the gastrointestinal system is one the first physiological systems affected by the ingestion of capsaicin or its analogs.

Lastly, the pharmacological tools involve the administration of TRPV1 agonists and antagonists. Both endogenous (i.e., anandamide or oleoylethanolamide) and exogenous vanilloids (i.e., capsaicin or RTX, a potent agonist naturally found in the plant genus *Euphorbia*) can be administered as TRPV1 agonists. Furthermore, several TRPV1 antagonists were recently developed by the pharmaceutical industry because of the capacity of such compounds to alleviate pain. Marked technological advancements have been made in this field recently as antagonists that selectively block a specific mode of TRPV1 activation have become available to researchers.

In addition to the administration of antagonists, an alternative method to investigate the physiological function of the TRPV1 channel is to desensitize these channels or the neurons that express them. This desensitization is a chronic phenomenon in which animals stop responding to capsaicin or RTX after the administration of repeated and/or large doses of these drugs (17). Capsaicin is a neurotoxin that destroys nociceptor neurons when administered in high doses. Caterina et al. (19) showed that sensory neurons as well as other TRPV1-expressing cells die after a few hours when treated with capsaicin, and Yamashita et al. (39) observed a reduced number of small diameter sensory fibers in the dorsal root ganglion of capsaicin pre-treated animals.

Having described the available tools for studying the physiological roles of the TRPV1 channel, the following sections will focus on the association between this channel and spontaneous locomotor activity and physical (aerobic) performance, as well as on the TRPV1 involvement in several physiological responses induced by physical exercise.

## Spontaneous physical (locomotor) activity

Spontaneous physical activity is fundamental to the sustainment of life and is positively correlated with physical fitness as the intensity, duration, and frequency of movements increase (40). In rodent experiments, spontaneous locomotor activity is measured in the home cage of the animal or during behavioral assessment tests (Table 1). Thus far, no study has investigated the possible link between spontaneous physical activity and the TRPV1 channel in humans.

**Table:**
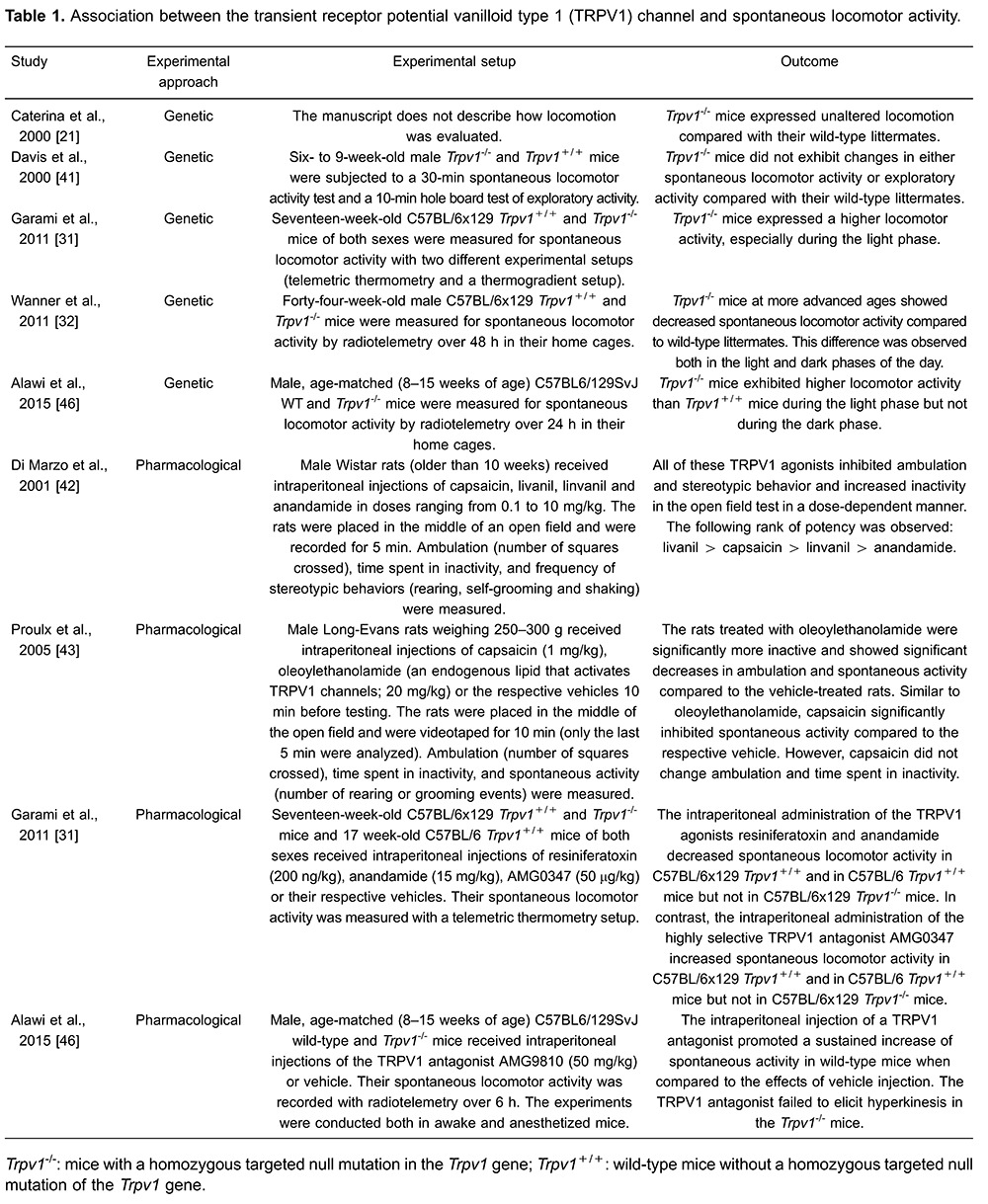

The first studies on this topic showed that the deletion of the *Trpv1* gene did not change the locomotion of rodents (21,41). However, all of these studies used brief observation periods, sometimes as short as 15 min. By recording spontaneous physical activity throughout the day, Garami et al. (31) observed that knockout mice had higher locomotor activity than that of their wild-type littermates. This finding in knockout mice was confirmed by the use of pharmacological tools; the intraperitoneal administration of RTX (an exogenous TRPV1 agonist) or anandamide (an endogenous TRPV1 agonist) attenuated the stress-induced hyperactivity in mice (31), confirming the reported hypokinetic action of these and other TRPV1 agonists in different tests (42–45). Garami et al. (31) also showed that the locomotor activity response was unaffected by these two TRPV1 agonists in knockout mice, thereby confirming that the anti-hyperkinetic effect of either agonist occurs via an action on TRPV1. In addition, the authors reported that a non-stressful intraperitoneal administration of AMG0347, a highly potent and selective TRPV1 antagonist, increased locomotor activity, an effect that was not observed in knockout mice. The hyperkinesis induced by an acute injection of a TRPV1 antagonist was reproduced recently (46), even when the drug (AMG9810) was injected into mice under stressful conditions. Together, these results support the hypothesis that the TRPV1 channel tonically suppresses general locomotor activity. It should be stated that the augmented locomotor activity caused by TRPV1 antagonists appears to be implicated in the genesis of the hyperthermia side-effect induced by these drugs (see the section on thermoregulation). As evidenced by Alawi et al. (46), no hyperthermic effect was observed after treatment with a TRPV1 antagonist when mice were anesthetized and thus unable to move.

**Figure 1:**
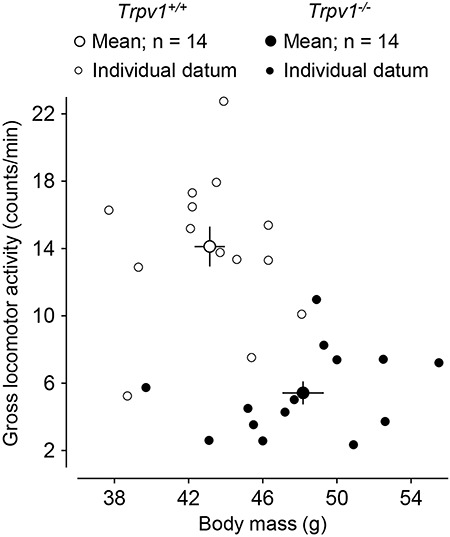
Transient receptor potential vanilloid type 1 (TRPV1) channel modulates spontaneous locomotor activity. The figure shows that aged *Trpv1*-knockout mice have a higher body mass and lower spontaneous locomotor activity than their genetically unaltered littermates. The figure was modified from Wanner et al. (32).

Quite unexpectedly, older knockout mice showed an opposite behavior, that is, decreased spontaneous locomotor activity compared with that of their age-matched wild-type littermates, which indicates that the TRPV1-mediated regulation of locomotor activity is age-dependent. Moreover, the decreased physical activity may help explain the exaggerated increase in the body mass of the knockout mice as they age (32) (Figure 1).

## Physical (aerobic) performance

Because the TRPV1 channel is highly expressed in nerve endings and in peripheral tissues, this channel is most likely involved in the regulation of several physiological functions, including thermoregulatory (47), cardiovascular (30,48) and metabolic functions (38). The TRPV1 channel also plays a role in systemic inflammation (49) and protects the gastrointestinal tract against inflammatory or functional gastrointestinal diseases (28). Together, these TRPV1-mediated functions (which are discussed in detail in the subsequent sections) may affect the physiological response to exercise and, therefore, influence fatigue and physical performance.

The role of the TRPV1 channel in the modulation of physical performance is still unclear; reports indicate that TRPV1 activation improves or does not change aerobic performance (Table 2). Luo et al. (34) observed an increase in the exercise performance of rats subjected to treadmill running after a 3-month treatment with dietary capsaicin. Similarly, Kim et al. (50) and Oh et al. (51) showed that a single oral administration of capsaicin increased the swimming endurance in mice. In agreement with the latter findings, TRPV1-desensitized rats displayed impaired performance during treadmill running. Dousset et al. (52) showed a shorter time to fatigue in adult rats that had received neonatal treatments of capsaicin compared with rats that received neonatal treatments of vehicle. However, Trudeau and Milot (53) observed no differences in swimming performance between TRPV1-desensitized and control rats. As in the study of Dousset et al. (52), the rats were desensitized neonatally and tested as adults. The different outcomes observed in these studies may result from type of exercise (treadmill running *vs* swimming), the animal species (rats *vs* mice) and the experimental approach for assessing TRPV1 function (pharmacological *vs* nutritional tools). Recently, more selective pharmacological tools (e.g., new TRPV1 antagonists) have been developed, allowing a more precise investigation of the physiological functions mediated by the TRPV1 channel. Therefore, future studies should be performed to enhance our understanding of why the activation of these channels produces different modulatory effects on spontaneous physical activity (decrement) and physical exercise performance (improvement). Interestingly, no study has addressed the relationship between TRPV1 and physical performance in humans.

**Table:**
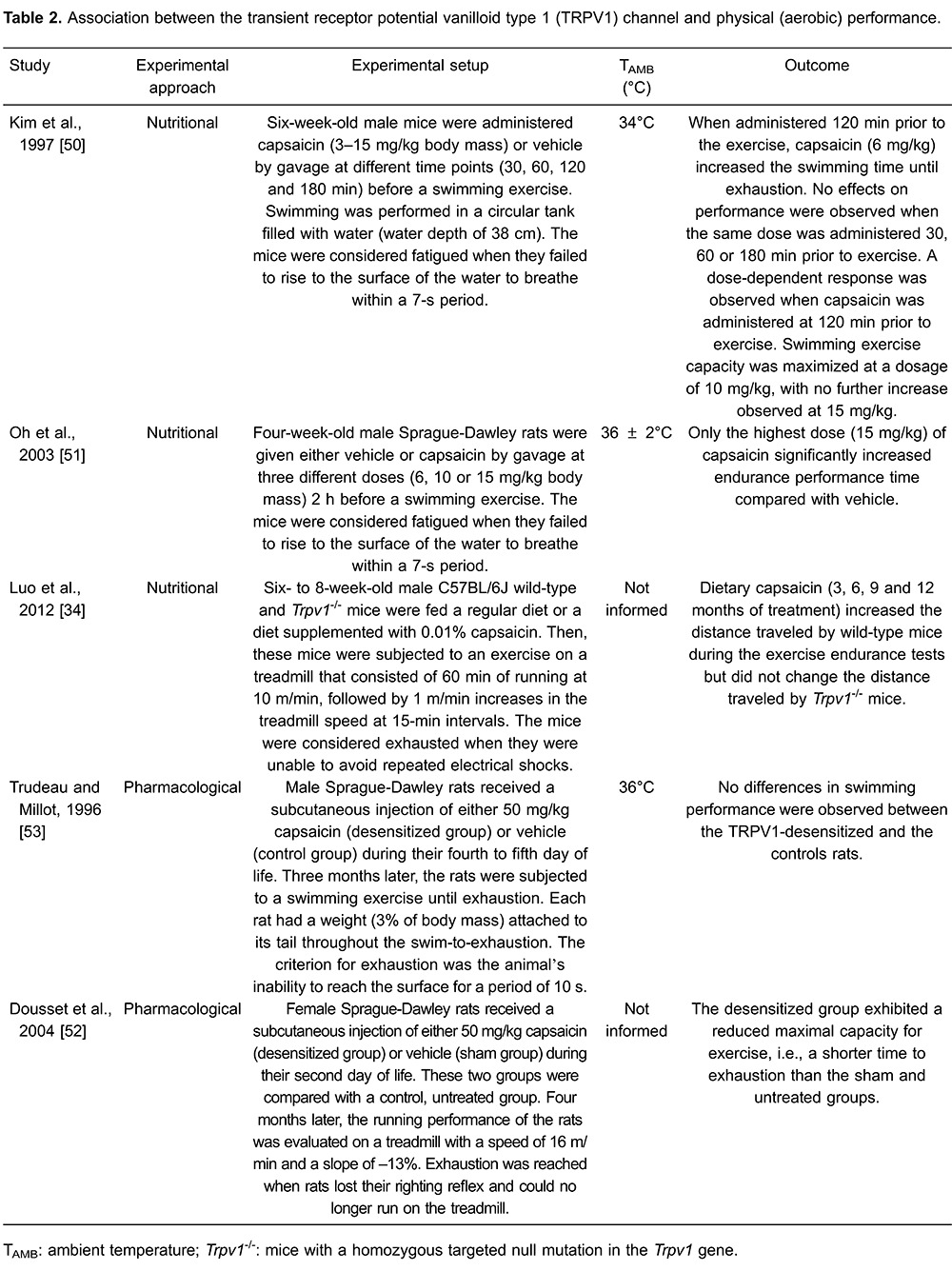

## Thermoregulation

Although the molecular structure of the TRPV1 channel was identified in 1997, the effects of capsaicin (i.e., an exogenous vanilloid) on body temperature have been known for several decades (54). The acute systemic administration of an exogenous vanilloid (either capsaicin or RTX) decreases the core body temperature (54,55) by increasing the cutaneous heat loss (54,56,57) and by promoting behaviors aimed at decreasing body heat conservation, such as the selection of an environment with lower temperature (55). After this initial hypothermic effect, the energy expenditure subsequently increases, which is likely due to a neuroendocrine counter-regulatory response involving the activation of the sympathetic nervous system and the hypothalamic-pituitary-adrenal axis (58). The physiological effects induced by capsaicin and RTX are not observed in mice with a genetic deletion of the TRPV1 channel (Trpv1-knockout mice), indicating that these channels mediate the physiological responses elicited by both exogenous vanilloids (21).

**Figure 2:**
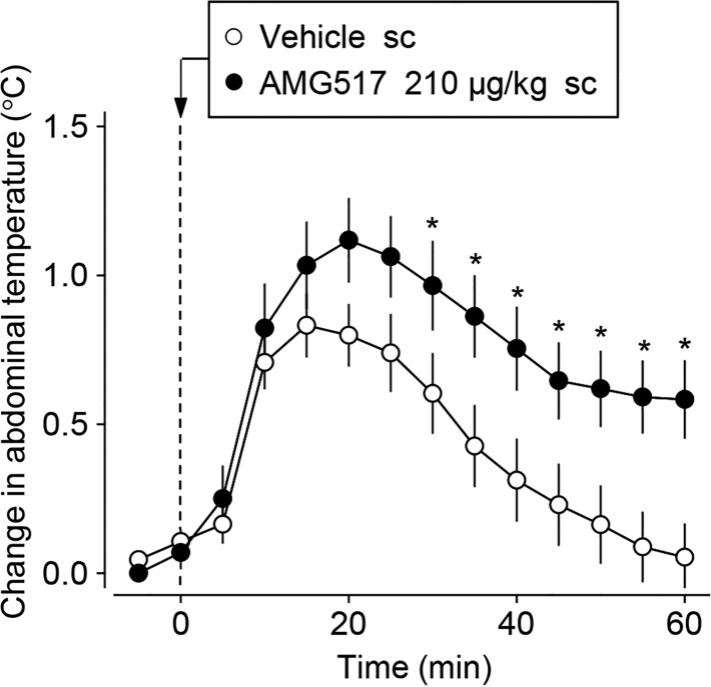
Systemic administration of a TRPV1 antagonist induces hyperthermia. The figure shows that subcutaneous injection of AMG517 (210 μg/kg, *sc*) increases the abdominal temperature of young mice maintained at 28°C. *Significant difference (P<0.05) compared to vehicle. TRPV1: transient receptor potential vanilloid type 1. The figure was modified from Wanner et al., 2012 (78).

The studies on the role of the TRPV1 channel in the modulation of physiological responses have drawn the attention of many researchers due to the role of this channel in the modulation of pain (21,59 – 61). For example, TRPV1 activation by the acute administration of exogenous agonists caused behaviors associated with pain and inflammation at the injection site (21). Thus, TRPV1 antagonists were and are being developed as powerful analgesics (59,60 [Bibr B61]); clinical trials indicated that the pharmacological blockade of TRPV1 channels produces analgesia after the extraction of a molar tooth, which represents an experimental model for the acute pain caused by surgery in humans. However, all of the subjects that participated in these clinical trials showed long-lasting hyperthermia, with one of these participants showing core temperature values higher than 40°C (62). This hyperthermia side effect was reproduced in several animal species (including mice; Figure 2) and results from decreased cutaneous heat loss and increased thermogenesis (46,47,63), suggesting that the TRPV1 channel is tonically active *in vivo* to regulate core temperature. This tonic activation inhibits heat production/conservation and activates heat loss pathways, thus contributing to the maintenance of core temperature within the normal, resting range (17,47). Therefore, the administration of an antagonist removes the tonic TRPV1 action on the thermoregulatory effectors, thereby causing hyperthermia (47).

To date, these side effects have limited the use of TRPV1 antagonists for the treatment of pain. It is likely that the unwanted hyperthermic effect depends on the different factors associated with channel activation, i.e., the potency of an antagonist in causing hyperthermia is related to the potency of the drug in blocking a certain mode (or modes) of TRPV1 activation (60). Evidence indicates that the administration of TRPV1 antagonists that do not block the proton-mode of activation but do block the activation by capsaicin and high temperature causes analgesia without triggering the unwanted hyperthermic effect (59,60).

The hyperthermia mediated by the administration of antagonists that block all of the modes of TRPV1 channel activation, such as AMG9810 and AMG0347, is an on-target effect because hyperthermia is not observed in Trpv1-knockout mice treated with these antagonists (31,46,47). A recent study provided evidence that TRPV1 controls resting thermoregulation by acting upstream in the activation of the sympathetic nervous system (46). Acute removal of the TRPV1 regulatory activity *via* the administration of an antagonist results in increased sympathetic outflow, characterized by higher levels of noradrenaline in the brown adipose tissue and the circulation (46), and this increased sympathetic outflow most likely favors the manifestation of hyperthermia. Interestingly, Alawi et al. (46) reported that deletion of the *Trpv1* gene results in a compensatory suppression of sympathetic activity and associated thermoregulatory pathways, which may explain the normal basal temperature of the knockout mice.

The involvement of the TRPV1 channel in thermoregulation is also supported by studies that employed techniques aimed at desensitizing this channel. As a consequence of the TRPV1 desensitization caused by pretreatment with capsaicin, rats showed exaggerated increases in core temperature during exposure to a hot environment (54,57). This inability to regulate the core temperature during heat exposure is associated with altered activity of the autonomic and behavioral thermoeffectors (4,54,57). Ob–l et al. (64) showed that desensitized animals exhibit lower cutaneous vasodilation in response to body heat storage, a failure to increase the grooming behavior during heat exposure, and a preference for a warm rather than a cold environment, even in situations of increased core temperature. In addition, Jancsó-Gábor (54) showed that the desensitized animals salivate less and, therefore, experience less evaporative heat loss when exposed to heat. However, Dib et al. (65) observed no differences in the cold-air requirements of desensitized and control animals during exposure to a hot environment. Taken together, these findings suggest that the TRPV1 channel modulates the recruitment of autonomic and some behavioral thermoeffectors in heat-exposed animals. Future studies should investigate the TRPV1-mediated recruitment of autonomic and behavioral thermoeffectors during physical exercise, a condition in which heat is markedly produced by metabolism and not necessarily gained from the environment.

## Metabolism

TRPV1 is involved in metabolism (14,58). Evidence indicates that Trpv1-knockout mice become obese as they age (31,32), although this result is not a universal observation (66). Haramizu et al. (37) reported that the oral treatment of mice for two weeks with capsiate promoted an improvement in energy metabolism and an inhibition of body fat accumulation, which are quite similar to the effects induced by regular swimming. In fact, the ingestion of TRPV1 agonists increases the lipolysis rate in exercising humans (38,67) without changing the plasma concentrations of epinephrine or norepinephrine (38). Along similar lines, mice treated with capsaicin exhibited higher plasma glucose and free fatty acid concentrations at rest (50) and after swimming (51). Nevertheless, measurements of the plasma catecholamine concentrations in exercising animals do not agree with the results of the previous study in humans. The plasma epinephrine and norepinephrine levels were higher in the control than in the TRPV1-desensitized rats that were subjected to exhausting exercise (53) and were higher in mice orally treated with capsaicin than in mice treated with placebo at the end of an exhausting swimming exercise (51). In summary, these results suggest that TRPV1 activation leads to increases in energy expenditure and lipolysis rate in exercising humans and rodents and thereby to sympathoadrenal excitation, at least in rodents.

## Cardiovascular system

The cardiovascular system is another important physiological system for exercise performance that may be regulated by the TRPV1 channel. Recent studies aimed to investigate the relationship between the capsaicin receptor and cardiovascular responses (29,30,47). Steiner et al. (47) and Sun et al. (30) observed that systemic activation of the TRPV1 channel by the administration of capsaicin reduces the mean arterial pressure, the heart rate and renal sympathetic activation. When the animals were previously treated with a potent TRPV1 antagonist, the cardiovascular effects promoted by capsaicin were prevented, except for a brief reduction in the heart rate (30). Furthermore, the TRPV1 channel is present in afferent elements of the baroreflex pathway; these channels are expressed in aortic baroreceptors, nodose ganglion neurons and the nucleus of the solitary tract (30). Additionally, the latter investigators demonstrated that systemic TRPV1 desensitization impairs the reflex control of blood pressure.

The TRPV1 channel is also involved in the exercise pressor reflex. Smith et al. (48) reported that TRPV1 blockade attenuates the increase in blood pressure during isometric contractions. Recently, TRPV1 expression was found in the vascular endothelium and smooth muscles (29). The activation of TRPV1 channels located in the vascular endothelium produces vasodilation by increasing the phosphorylation of endothelial nitric oxide synthase (68,69). Moreover, dietary capsaicin enhances TRPV1 expression in endothelial cells, and this response is associated with an increase in endothelium-dependent vasodilation (68). The activation of the TRPV1 channels also induces vasodilation via increased release of the calcitonin gene-related peptide at the nerve terminals of capsaicin-sensitive neurons that innervate peripheral arteries (70). This vasodilation seems to mediate the decrease in blood pressure observed after the administration of TRPV1 agonists. Therefore, it is likely that TRPV1 activation changes the reflex regulation of blood pressure and the vasomotor tone, thereby changing the cardiovascular response during physical efforts. In addition, we assume that physical training may modulate the expression of these channels, as indicated in a study conducted with animals that were experimentally subjected to heart failure; aerobic training partially reversed the reduced TRPV1 expression in the dorsal root ganglia and partially restored the response of group IV afferents to an intra-arterial injection of capsaicin (71).

## Gastrointestinal system

The TRPV1 channel may also modulate aerobic performance and physiological responses to exercise by affecting gastrointestinal function. Gastrointestinal symptoms have been observed in 30–50% of athletes competing in prolonged events, such as marathon runners and cyclists (72), and have been observed in up to 93% of participants in a long-distance triathlon (73). Overall, although these symptoms are not severe, they may compromise sport performance by reducing exercise intensity or even ceasing the exertion during a competition (73). In fact, physical exercise under conditions of environmental heat stress leads to increases in intestinal permeability and in the bacterial content in the blood and liver of mice (74). Augmented intestinal permeability may be one of the causes of the surge in gastrointestinal symptoms.

Although capsaicin receptors are mainly located in neurons, these receptors were identified in non-neuronal cells of the digestive tract of various animal species, including humans. Cells with immunoreactivity to TRPV1 were identified in the epithelial tissue of the esophagus and stomach and in the small bowel mucosa (75,76). The TRPV1 channel is likely important in tissue protection and in the restoration of epithelial tissue because changes in TRPV1 expression are observed in response to several inflammatory or functional gastrointestinal diseases (28). The capsaicin receptor protects the gastrointestinal barrier function, thus preventing the increase in permeability (17,62). These protective effects result from the modulation of the host immune response (77) and the regulation of local blood flow (78) and the cellular ionic medium (78). The latter mechanism is supported by observations showing that changes in ion flux influence the binding of tight junctions (79). It is therefore likely that TRPV1 channels help to maintain the integrity of the gastrointestinal barrier during exercise.

## Inflammation

The TRPV1 channel is critically involved in the systemic responses such as augmented energy expenditure and water retention that favor inflammation (58). TRPV1 is stimulated by a whole range of pro-inflammatory factors (58), and the injection of TRPV1 agonists (either capsaicin or RTX) induces pain at the injection site (21). In addition, there is evidence that gastrointestinal inflammation causes hyperalgesia at least in part by the upregulation and sensitization of TRPV1 (28), and that TRPV1 desensitization changes the release of TNF-α during sepsis (80). However, the induction of a pro-inflammatory response after the activation of the TRPV1 channel is not a universal finding (49). For instance, the administration of a TRPV1 antagonist (capsazepine) increased mortality in rats treated with a shock-inducing dose of lipopolysaccharides (81); of note, the administration of high doses of lipopolysaccharides represents an experimental model that produces an exaggerated systemic inflammatory response syndrome that usually causes animal death. Thus, at least in rodents undergoing aseptic systemic inflammation, the TRPV1 channel appears to play an anti-inflammatory (regulatory) role. According to Holzer (28), the observations that TRPV1 stimulation either reduces or exaggerates tissue inflammation may reflect stimulus- and tissue-dependent differences in the process of neurogenic inflammation. It is relevant to point out that the role of TRPV1 in inflammation may be influenced by aging because the effect of a TRPV1 antagonist (AMG517) on the inflammatory response caused by a shock-inducing dose of lipopolysaccharides in aged mice was the opposite to that observed in young mice (77).

Inflammatory responses may be indirectly linked to physical performance. It has been proposed that part of the increase in core temperature observed during exercise is caused by the augmented production of pro-inflammatory cytokines that results from the increased core temperature. This mechanism could generate a cyclical phenomenon, that is, increases in core temperature augment bacterial translocation through the gut wall, which in turn promotes systemic inflammation and ultimately further increases the core temperature (73,82). Jeukendrup et al. (73) and Bradford et al. (82), respectively, showed that in humans, the plasma concentrations of IL-6 and TNF-α increased during prolonged exercise. This increase in circulating cytokines may enhance exercise hyperthermia through the stimulation of the synthesis and release of prostaglandin E_2_ (PGE_2_) by the brain (83). In fact, the administration of a PGE_2_ synthesis inhibitor mitigated exercise-induced hyperthermia in humans (82). Similarly, heat strain was increased during endurance exercise in the heat that was conducted 30 min after a muscle-damaging eccentric exercise, which increased the circulating levels of IL-6 (84). Thus, it is likely that the TRPV1 channel changes the inflammatory status during exercise and changes thermoregulatory responses, partially by modulating the release of pro-inflammatory cytokines.

## Water and electrolyte balance

Aside from the aforementioned physiological functions, the TRPV1 channel is also involved in the regulation of plasma volume and osmotic balance. Increases in the core temperature induce thermoregulatory responses that promote water loss, generating internal hyperosmolar conditions (58). In this context, the TRPV1 channels in the hypothalamus assist in the regulation of plasma volume by promoting water retention (85). This activation of hypothalamic capsaicin receptors may become important during physical exertion, when the core temperature is usually increased and water is lost to facilitate evaporative heat loss from the body.

## Muscular hypertrophy and mitochondrial biogenesis

TRPV1 channels may also participate in some chronic adaptations induced by regular physical exercise or training programs, including muscular hypertrophy and mitochondrial biogenesis. The TRPV1 channels are expressed in human skeletal muscle (86). As suggested by studies conducted using mice and rats, these channels are expressed in the sarcoplasmic reticulum membrane but not in the sarcolemma (27,87). The activation of the capsaicin receptor promotes the release of calcium from the sarcoplasmic reticulum, which in turn stimulates the mammalian target rapamycin, a molecular mediator that promotes muscle hypertrophy. These findings suggest that the effects induced by capsaicin administration are comparable to the effects induced by mechanical overload (88). Collectively, these results indicate the potential of TRPV1 agonists in the development of ergogenic strategies and for reversing clinical conditions, such as muscle atrophy.

In addition to muscular hypertrophy, mitochondrial biogenesis is also likely altered by the chronic administration of capsaicin. The *in vitro* administration of capsaicin promoted the expression of peroxisome proliferator-activated receptor gamma co-activator-1α (PGC-1α) in C2C12 myotubes (34). Moreover, PGC-1α was upregulated *in vivo* in skeletal muscle by dietary capsaicin-induced TRPV1 activation or genetic overexpression of TRPV1 in mice. In addition, TRPV1 activation increased the expression of genes involved in fatty acid oxidation and mitochondrial respiration, promoted mitochondrial biogenesis and increased the number of oxidative fibers. These beneficial adaptations promoted by dietary capsaicin prevented high-fat diet-induced metabolic disorders (34).

## Final remarks

**Figure 3:**
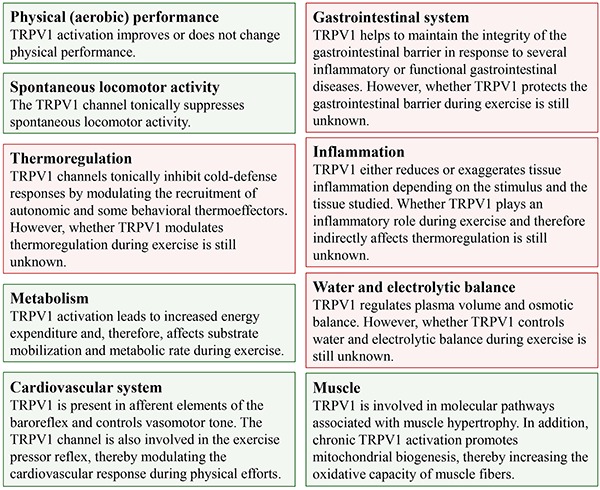
Overview of the physiological functions exerted by transient receptor potential vanilloid type 1 (TRPV1) channels. The green boxes indicate responses that are mediated by TRPV1 channels during physical activity or exercise, whereas the red boxes indicate TRPV1-mediated physiological responses that have not been investigated under such conditions.

In addition to nociception and thermoregulatory responses, new functions of the TRPV1 channel have been revealed by recent studies; these functions are associated with the modulation of spontaneous physical activity and the regulation of muscle homeostasis, metabolism, osmolality and immune responses. Some of these TRPV1-mediated effects have already been observed during exercise, such as increased energy expenditure and lipolysis rate and the modulation of the exercise pressor reflex. The TRPV1-mediated modulation of several physiological responses is summarized in Figure 3.

This broad range of physiological functions controlled by the TRPV1 channel suggests that TRPV1 activation regulates physical (aerobic) performance. In addition, the data presented in this review indicate new possibilities for investigating the role of the TRPV1 channel in the acute effects induced by a bout of physical exercise and in the chronic effects induced by physical training.
